# Decorin Protein Core Affects the Global Gene Expression Profile of the Tumor Microenvironment in a Triple-Negative Orthotopic Breast Carcinoma Xenograft Model

**DOI:** 10.1371/journal.pone.0045559

**Published:** 2012-09-19

**Authors:** Simone Buraschi, Thomas Neill, Rick T. Owens, Leonardo A. Iniguez, George Purkins, Rajanikanth Vadigepalli, Barry Evans, Liliana Schaefer, Stephen C. Peiper, Zi-Xuan Wang, Renato V. Iozzo

**Affiliations:** 1 Department of Pathology, Anatomy and Cell Biology, and the Cancer Cell Biology and Signaling Program, Kimmel Cancer Center, Thomas Jefferson University, Philadelphia, Pennsylvania, United States of America; 2 LifeCell Corporation, Branchburg, New Jersey, United States of America; 3 Roche NimbleGen, Inc., Research and Development, Madison, Wisconsin, United States of America; 4 Department of Pharmacology, Goethe University, Frankfurt, Germany; National Cancer Institute at Frederick, United States of America

## Abstract

Decorin, a member of the small leucine-rich proteoglycan gene family, exists and functions wholly within the tumor microenvironment to suppress tumorigenesis by directly targeting and antagonizing multiple receptor tyrosine kinases, such as the EGFR and Met. This leads to potent and sustained signal attenuation, growth arrest, and angiostasis. We thus sought to evaluate the tumoricidal benefits of systemic decorin on a triple-negative orthotopic breast carcinoma xenograft model. To this end, we employed a novel high-density mixed expression array capable of differentiating and simultaneously measuring gene signatures of both *Mus musculus* (stromal) and *Homo sapiens* (epithelial) tissue origins. We found that decorin protein core modulated the differential expression of 374 genes within the stromal compartment of the tumor xenograft. Further, our top gene ontology classes strongly suggests an unexpected and preferential role for decorin protein core to inhibit genes necessary for immunomodulatory responses while simultaneously inducing expression of those possessing cellular adhesion and tumor suppressive gene properties. Rigorous verification of the top scoring candidates led to the discovery of three genes heretofore unlinked to malignant breast cancer that were reproducibly found to be induced in several models of tumor stroma. Collectively, our data provide highly novel and unexpected stromal gene signatures as a direct function of systemic administration of decorin protein core and reveals a fundamental basis of action for decorin to modulate the tumor stroma as a biological mechanism for the ascribed anti-tumorigenic properties.

## Introduction

The traditional view of tumorigenesis has long been considered in the perspective of oncogenes and tumor suppressor genes governing diverse cellular processes such as cell proliferation, survival, migration, and metastasis, with a prominent focus on understanding the cellular imbalance existing among these two populations of nuclear encoded genes. However, the importance of the surrounding tumor environment has begun to emerge as an active participant in orchestrating many aspects of tumor growth and progression including invasion, metastasis, and angiogenesis [1], [2]. The heterogeneous interactions between neoplastic cells and stromal components such as fibroblasts, tumor macrophages, extracellular matrix, host and tumor endothelial cells as well as basement membrane, collectively known as the tumor microenvironment, can have profound effects on tumorigenesis. However, despite the importance of tumor-stroma interactions, there is a limited understanding of its composition and how the complex inter-relationships between growing malignant cells and stromal constituents take place.

The stromal-specific decorin is encoded by a large and complex gene (*DCN*) located on chromosome 12q23 which contains two alternatively-spliced leader exons [3] and a quite complex promoter region [4], [5]. Notably, *DCN* transcriptional activity is induced by hypomethylation of its promoter [6], [7] and by quiescence [8], [9], and inhibited by TNFα [9], TGFβ [10] and viral oncogenes [11]. Decorin is a secreted small leucine-rich proteoglycan that binds collagen I and regulates fibrillogenesis [12]–[23], and is known to bind avidly to TGFβ [24] and regulate its bioactivity [25]–[27]. In addition, decorin-deficient mice are less susceptible to experimental infection by *Borrelia burgdorferi*, the spirochete responsible for Lyme disease in humans [28], [29].

When in soluble form, decorin inhibits tumor growth by downregulating several receptor tyrosine kinases (RTKs) [30] such as the EGFR [31]–[34], IGF-IR [35]–[38], and Met [39]–[41] primarily by evoking caveolin-mediated internalization and degradation [31], [39], [42]. This leads to mobilization of intracellular calcium [43], concurrent induction of cyclin-dependent kinase inhibitor p21^WAF1^ [44], [45] and subsequent degradation of key downstream effectors such as β-catenin and Myc in various tumor xenografts [40].

Under growth conditions, β-catenin and Myc are stabilized by activation of Wnt and various RTK signaling pathways, which are often hyperactive in cancer. Thus, upstream activation of these signaling pathways leads to inactivating phosphorylation of GSK-3β by PI3K/Akt to allow for translocation of active β-catenin and Myc into the nucleus where, through association with various nuclear co-activator complexes, enable induction of a large number of genes including Myc itself [46], [47], and AP4, a transcriptional repressor of p21 [48]. In contrast, decorin, by neutralizing the activity of EGFR and Met, relieves GSK3β inactivation, and leads to non-canonical GSK3β-evoked phosphorylation of β-catenin as well as Myc at Thr58 [39], [40], thus leading to 26S proteasomal degradation. Thereby, the synthesis of AP4 would cease and p21 would be released from Myc/AP4-mediated transcriptional repression, as a direct biological manifestation of decorin-evoked suppression of RTK activity and tumorigenic growth [49]. Moreover, the relevance of decorin in cancer progression has been demonstrated in mutant mice where ∼30% of decorin-null mice develop spontaneous intestinal tumors [50], whereas decorin-null mice carrying a targeted disruption in p53 succumb within 3–4 months to aggressive lymphomas [51].

Decorin is also involved in the regulation of angiogenesis [52], [53] and blocks tumor cell-mediated angiogenesis by downregulating VEGFA production [54], as well as Met and downstream angiogenic networks [55]. Moreover, recombinant decorin proteoglycan or decorin protein core inhibits metastatic spreading of breast carcinoma xenografts [56], [57]. Adenoviral-mediated decorin gene delivery and/or via systemic treatment retards the growth of various tumor xenografts including squamous, breast, and prostate carcinomas [32], [42], [56]–[62]. Low levels of decorin are present in invasive breast carcinomas [63] and this trait is associated with poor outcome in breast [64] and lung cancer patients [65]. Moreover, abnormal decorin expression has been detected in mammographic density [66], a major risk factor for breast cancer [67]–[69], and array data have demonstrated decorin upregulation during mammary gland involution, likely contributing to increased collagenization [70]. Thus, the expression and/or activity of decorin could affect breast cancer risk. Accordingly, any mechanism that would boost endogenous expression of decorin [71] or any therapeutic modality that could efficiently and specifically deliver decorin to carcinomas could represent a novel therapeutic choice against cancer [72], [73].

Thus, we wished to deepen our molecular understanding of its potential role in modulating the tumor stroma by directly investigating, at high resolution, the gene expression signature of a triple negative breast carcinoma microenvironment. Therefore, for the first time, we were able to dissect the complex inter-relationship present between the tumor and the stroma by utilizing a novel high-density microarray platform capable of simultaneously measuring expressed transcripts derived from both the mouse (i.e. the stroma) and the neoplasm (i.e. the human tumor cell line, MDA-MB-231) via interrogation of orthotopic tumor xenografts treated systemically with decorin.

In this study, we report the stromal gene expression signature obtained with systemic delivery of decorin protein core and discover a total of 374 genes, which showed differential expression profiles. Further, and unexpectedly, we found by DAVID gene ontology analysis, a predominant inhibition of inflammatory and immune response genes with concurrent induction of various tumor suppressors and cellular adhesion molecules. Moreover, independent verification of the top 12 genes demonstrating >2-fold up or down regulatory changes identified *Mrgpra2, Siglech, Irg1* and *Il1b* to be potently suppressed concurrent with *Zc3hav1, Peg3*, and *Bmp2k* induction. These data posit for decorin protein core as a potent tumor repressor by attenuating inflammation and metastasis, which constitute several hallmarks of cancer.

## Results

### MDA-231(GFP+) Tumor Xenografts Respond to Systemic Delivery of Decorin

**Figure 1:**
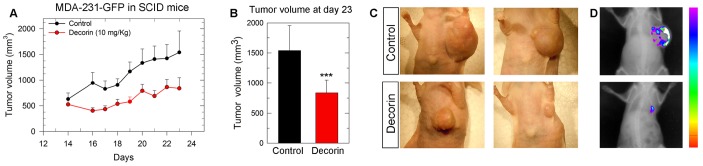
Systemic delivery of decorin protein core inhibits the growth of orthotopic breast carcinoma xenografts. **A:** Growth of MDA-231(GFP+) xenografts following daily i.p. injections of either PBS (black circle, control) or recombinant decorin protein core (red circle, 10 mg/kg). **B:** Tumor volumes at day 23, ***P<0.001. **C:** Representative macroscopic photographs of control and decorin-treated animals at day 23. **D:** Bioluminescence superimposed on x-ray analysis of a representative control and decorin treated animal at day 23. Images were captured with a Kodak In-Vivo Multispectral Imaging System FX.

We performed experiments utilizing a very aggressive MDA-231(GFP+) triple-negative breast carcinoma cell line. We discovered that decorin evoked growth inhibition *in vitro*, enhanced apoptosis and blocked EGF- and FGF-mediated evasion from Matrigel (not shown), indicating that decorin exerts similar inhibitory activity on EGFR and Met dependent pathways as shown for other carcinoma cells [39], [40], [42], [57]. Subsequently, we injected ∼3 million MDA-231(GFP+) cells into the mammary fat pads of SCID mice (n = 6 each), and when the tumor reached palpable size we treated them with daily i.p. injections of recombinant decorin protein core (10 mg/Kg). Purity of the preparation of decorin protein core was established by SDS polyacrylamide gel electrophoresis followed by staining with Colloidal Coomassie blue which can detect as little as 5 ng of protein. We did not find any co-purifying contaminants using this method (Figure S1). Therefore, based on this information, and since the largest amount of decorin protein core loaded was 5 µg, our preparation of decorin protein core is >99% pure. The results show that decorin protein core (henceforth designated decorin) was capable of retarding the growth of the breast carcinoma xenografts (Figure 1A–D), which was highly significant at day 23 (Figure 1B, *P*<0.001). At the end of the experiments, the tumors were snap-frozen in liquid nitrogen and RNA was extracted for NimbleGen analysis.

### Analysis of Stromal (Mus Musculus) and Epithelial (Homo Sapiens) mRNA by the NimbleGen High-Density Mixed Expression Array Platform

**Figure 2:**
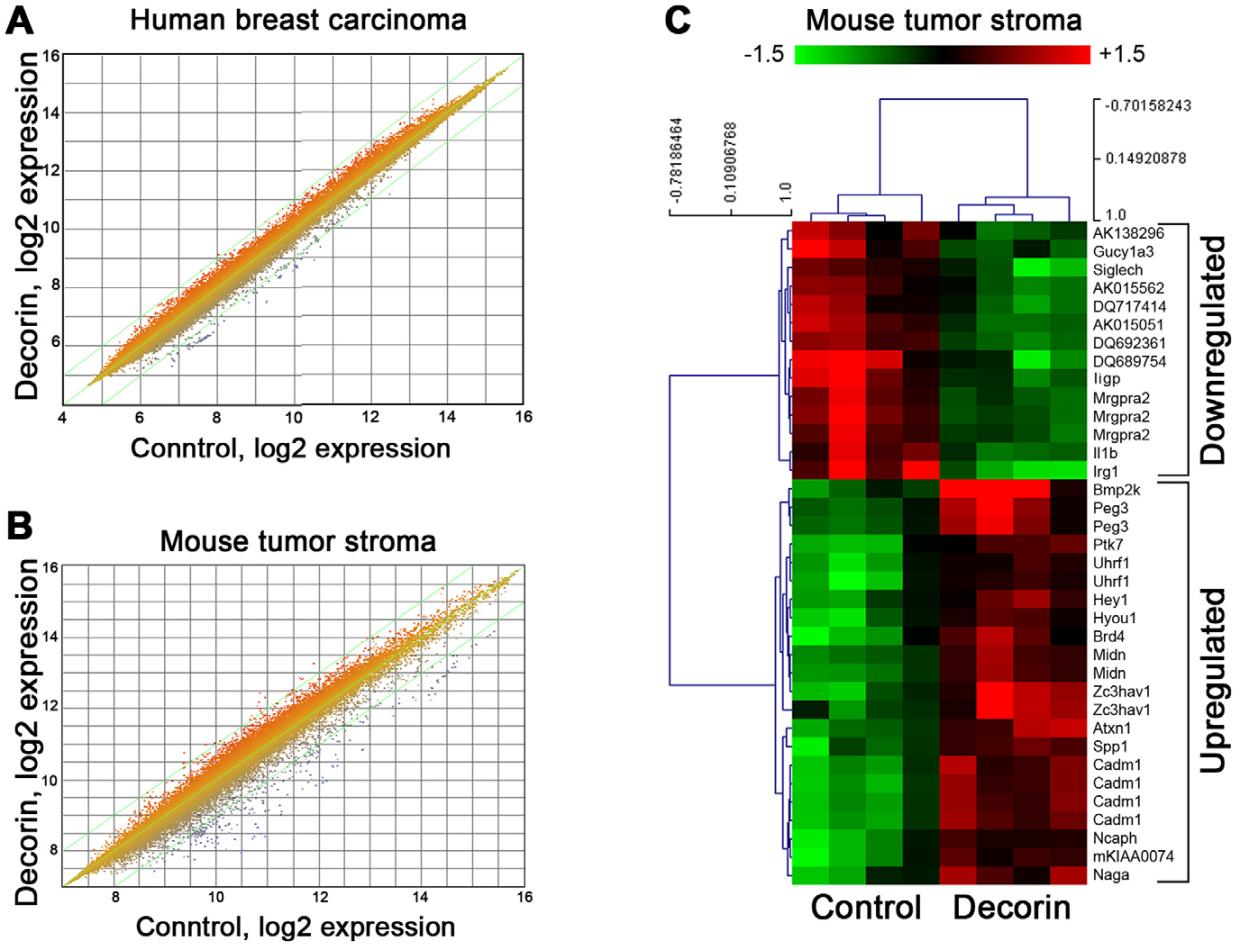
Systemic delivery of decorin protein core alters the gene expression profile of the mammary tumor stroma xenografts. **A,B:** Global expression profile comparing log2 expression of vehicle control vs. decorin-treated tumor xenograft samples. Notice that the effects of decorin are more pronounced in the gene expression profile of the mouse-derived tumor stroma. **C:** Hierarchical clustering of the top 36 genes downregulated (n = 12) or upregulated (n = 15) in the mouse tumor stroma in control and decorin-treated mice. These 27 top genes were selected on the basis of two criteria: >2-fold change and *P*<0.01.

To achieve a greater understanding of the governing interactions between the neoplastic cells and surrounding microenvironment we initially investigated the transcriptome of breast cancer stroma utilizing the orthotopic human mammary carcinoma xenografts described above. This approach is based on the utilization of a unique high-density microarray platform capable of differentiating and profiling transcripts from both human and mouse origin on the same array (NimbleGen 3×720 K). The mixed gene expression chip allows three arrays per slide with each array exhibiting 720,000 probes representative of transcripts originating from both human neoplastic cells and mouse stroma components. Appropriate sample labeling of the validated cDNA was followed by hybridization to the 3×720 K array followed by scanning using the Roche NimbleScan software, which normalizes the expression data using quantile normalization as determined by the Robust Multichip Average algorithm. Expression data were tested for significance using a mixed effects model ANOVA in R. Genes were sorted by *P*-value, and then by fold change. Genes with *P*<0.05 were placed into upregulated and downregulated groups as illustrated with the resultant heat maps. We have deposited the results of the microarray data sets in the publically-available GEO System with accession number GSE37937. Global expression profiles comparing human breast carcinoma in decorin treated when compared to vehicle control showed that most of the genes in two independent experiments (n = 6 each) showed close grouping with only minor changes (Figure 2A). Indeed, we found no significant (*P*<0.05) changes in any of the human-derived genes. In contrast, the number of genes derived from the tumor stroma of decorin-treated animals showed significant changes, with both upregulated and downregulated genes (Figure 2B). To further refine the search, we performed Principal Component Analysis and eliminated two outliers from each group. With this filtered set (n = 4 each), we identified 374 differentially expressed genes with *P*<0.05. Among all the genes, we selected the top 27 genes exhibiting greater than 2-fold down or upregulation and *P*<0.01. Notably, these genes clustered very well (Figure 2C).

These findings show that systemic delivery of decorin in this triple-negative orthotopic breast carcinoma xenograft predominantly affects the global gene signature of the tumor microenvironment.

### Stromal Decorin Differentially Inhibits Expression of Immunomodulatory Genes and Concomitantly Induces Expression of Mutliple Tumor Suppressor Genes

**Figure 3:**
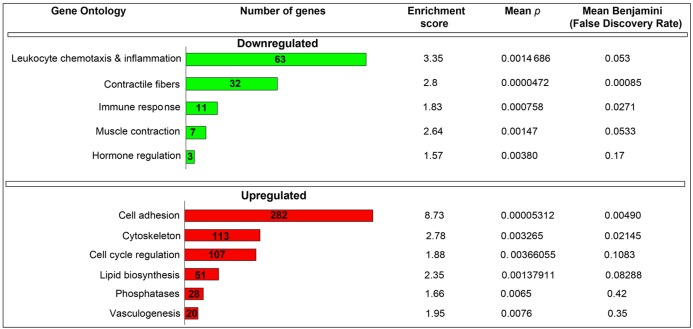
DAVID gene ontology analysis. Rigorous classification of the 374 differentially expressed genes within the tumor microenvironment of triple negative breast carcinoma xenografts following systemic treatment with decorin protein core for 23 days. Determinations for the down- and upregulated classes were selected on highly stringent criteria (*P*<0.05). Thus, all subsets above this value were analyzed by summing all genes within a specific gene ontology class followed by deriving the mean *P* and Benjamini values.

Gene ontology (GO) analysis was performed on the microarray data set comprising the 374 differentially expressed genes exhibiting *P*<0.05. Utilization of the Database for Annotation, Visualization, and Integrated Discovery (DAVID v6.7) [74] allowed for a detailed ontology analysis based on the assigned cellular and molecular functions of the differentially regulated stromal genes in the presence of decorin. We thereby found the genes clustering into 5 discrete downregulated and 6 upregulated GO terms (Figure 3), associating with high significance values.

Remarkably, the leukocyte chemotactic and inflammatory genes represented the most significantly downregulated class (n = 63, enrichment score of 3.35, *P* = 0.0014) among the differentially regulated stromal genes followed by contractile fiber genes and those responsible for orchestrating the general immune response (Figure 3). This representation of GO terms is appropriate and consistent as the most downregulated gene (−5.3 folds), as determined by microarray analysis (cfr. Figure 2C), was immune response gene 1 (Irg1). Interestingly, full expression of Irg1 is dependent on estrogen signaling [75] and is a gene typically induced in macrophages by pro-inflammatory stimuli such as TLR agonists (lipopolysaccharide), IFNγ, and TNFα [76], [77]. It is important to note that our MDA-231 orthotopic breast carcinoma models used for these studies are triple negative for ERα/PgR/HER2. Thus, these findings indicate an estrogen-independent mode of Irg1 downregulation via decorin. Importantly, and to the best of our knowledge, this gene has not previously been implicated in breast tissue and/or tumor progression and may serve as a novel component in tumorigenesis and detection.

Other suppressed immunomodulatory genes of interest included Siglech (Sialic acid binding Ig-like lectin; −2.3 folds, *P* = 0.00844), Lipg (IFNγ inducible GTPase; −2.72 folds, *P* = 0.0082), and Il1b (Interleukin 1β; −2.3 fold, *P* = 0.0046). Siglech is a member of the Ig gene superfamily, which binds avidly to sugars and is the mouse homologue of SIGLEC-15, the only known family member to maintain complete conservation throughout vertebrate evolution [78]. Lipg is a member of the small GTPase family of Golgi- and endoplasmic reticulum-associated subfamily activated primarily by TLR signaling and IFNγ [79]–[81]. Finally, Il-1β is a primary mediator of innate and adaptive immune responses and has been firmly established as a regulator of cancer progression via control of proliferation and apoptotic processes [82].

Taken together, this signature of decorin-suppressed stromal genes indicates an immunosuppressive nature and constitutes a very poorly understood and under-investigated area of decorin bioactivity. However, recent publications are now positing a potent immunomodulatory role for decorin in various settings [83], [84] and in tumorigenesis [85].

As for the stromal induced gene subsets, cell adhesion molecules commanded the majority as the most significantly enriched GO term (n = 282, enrichment score of 8.73, *P* = 0.00005) followed by cytoskeletal components (n = 113, enrichment score of 2.78, *P* = 0.0032), and cell cycle regulatory genes (n = 107, enrichment score of 1.88, *P* = 0.0036) (Figure 3). The cellular and molecular functions these gene subsets represent are highly consistent for the ascribed properties of decorin functioning in a tumor repressive capacity as these classifications are associated with tumor suppressive activity.

The top stromal upregulated gene was a kinase induced by Bmp2, known as Bmp2 inducible kinase (Bmp2k). Bmp2k is a largely unknown and poorly investigated nuclear-localized serine/threonine kinase that represents another candidate gene not yet implicated in human breast cancer and might represent an important target for decorin bioactivity as well as a potentially novel biomarker for tumor progression.

Another decorin-induced stromal gene was cell adhesion molecule 1 (Cadm1, −2.7 fold, *P* = 0.0011), a transmembrane glycoprotein containing Ig-like C2 modules in the ectodomain [86], also known as Tumor Suppressor in Lung Cancer (TSLC1). Expression of Cadm1 is lost in various malignancies, including breast [87], due to promoter hypermethylation.

An additional decorin-induced target classified as a tumor suppressor gene within the stroma, was Peg3 (paternally expressed gene 3, 2.49-folds, *P* = 0.0078) and it encodes a zinc finger transcription factor (Cys_2_His_2_ variety) of the Krüpple-type family. Similarly to Cadm1, this gene is also frequently silenced by promoter hypermethylation and/or loss of heterozygosity in several tumors including ovarian cancer [88] and gliomas [89].

The results derived from the DAVID gene ontology analysis provided a critical assignment of cellular and molecular function to our data subsets that are largely consistent with the top scoring genes as determined by the mixed microarray. These data suggest a novel role for decorin as a key immunosuppressive agent that is acting on the tumor stroma that may compromise the tumor-immune system interface, which is crucial for maintaining continued tumor progression. Simultaneously, decorin induces the expression of several tumor suppressor genes that, like several of the downregulated targets, have never previously been linked to cancer progression.

### Validation of Differentially-expressed Stromal Genes by Systemic Decorin Delivery

Independent verification of microarray datasets is absolutely critical for determining the validity and biological fitness of the presumed findings. Since the microarray platform was unique insofar as containing probes representing two different species and our data report the impact of decorin on stromal gene signatures (of *Mus musculus* origin) we therefore designed primers using the Universal Probe Library (Table S1) that are specific only for *M. musculus* transcripts, aimed at the top 6 up- and down-regulated (exhibiting *P*<0.01) stromal genes. Thereby, we have rigorously authenticated our primers in both *M. musculus* cDNA, harvested from NIH3T3 cells (positive control), and *Homo sapiens* cDNA as derived from HeLa cells (negative control for primer specificity). Our primers were capable of differentiating and recognizing only *M. musculus* mRNA species, in contrast to *H. sapiens* mRNA as validated by qPCR under identical thermal cycling conditions (Table 1). Further, eight of the twelve primer sets had no discernable amplification in *H. sapiens* when compared to *M. musculus* where a majority of the primer sets reported signal in the early to mid-range of thermal cycle 20. Thus, these data indicate high sensitivity with exquisite recognition and specificity for only *M. musculus* transcripts.

**Table 1:** Demonstration that qPCR verification primers exhibit exquisite specificity for NIH3T3 (mouse embryonic fibroblasts) cDNA when directly compared to HeLa (human squamous cell carcinoma) cDNA.

Official Gene Symbol	HeLa cDNA Average Ct (± S.D.)	NIH3T3 cDNA Average Ct (± S.D.)	Average ΔCt (HeLa – NIH3T3 Ct)
Bmp2k	32.92 (±0.18)	26.03 (±0.16)	6.89
Mrgpra2	36.57 (±0.36)	28.28 (±0.38)	8.29
Cadm1	Not Detected	37.40 (±0.16)	N/A
Hey1	Not Detected	31.52 (±0.49)	N/A
Gucy1a3	35.88 (±0.70)	28.33 (±0.27)	7.55
Peg3	Not Detected	25.41 (±0.17)	N/A
Brd4	37.25 (±0.33)	22.58 (±0.02)	14.67
Zc3hav1	Not Detected	25.36 (±0.20)	N/A
Siglech	Not Detected	29.67 (±0.12)	N/A
Ligp	Not Detected	33.17 (±0.14)	N/A
Irg1	Not Detected	34.83 (±4.26)	N/A
Il1b	Not Detected	26.49 (±1.00)	N/A

Average Ct (± S.D.) values were obtained via qPCR for each gene primer set listed above when exposed to either NIH3T3 or HeLa template cDNA following standard 40 cycle SYBR Green evaluation as performed on the Roche 480 LightCycler II platform in either quadruplicate (HeLa) or triplicate (NIH3T3). HeLa Ct values scored as “Not Detected” refers to either all four replicates failing to register a detectable amplicon threshold or reflects that only one replicate achieved threshold detection, which most likely reflects stochastic amplicon formation at higher Ct values (>35); further, values that surpassed a Ct of 35 were considered as “Not Detected”.

**Figure 4:**
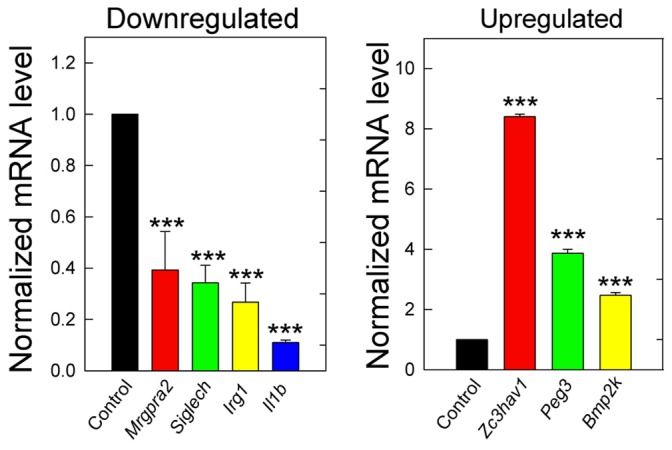
Independent qPCR verification of NimbleGen targets in orthotopic MDA-231 tumor xenografts following systemic delivery of human recombinant decorin protein core. Of the 36 top scoring candidates, we selected the top six-upregulated and downregulated targets and verified the corresponding expression patterns independently via real time quantitative PCR (qPCR) analysis on the same MDA-231 tumor xenograft total RNA utilized for the NimbleGen Mixed expression array. Since these 12 candidate genes represent differential stromal (mouse) gene expression signatures, we ensured our primers exhibit extreme stringency and specificity for only *Mus musculus* transcripts (*cfr.* Table 1). Therefore, we were able to demonstrate reproducibility and verification for seven of the twelve candidate genes, representing four downregulated (*Mrgpra2, Siglech, Irg1, Il1b*) and three upregulated (*Zc3hav1, Peg3, Bmp2k*) transcripts in our MDA-231 xenograft mouse model. Data are representative of three independent samples for each sample cohort in quadruplicate replicates, analyzed with the ΔΔCt method (please see Materials and Methods for a more detailed explanation), and reported as the average fold change ± SEM (***P<0.001).

Utilizing mRNA extracted from the same tumor xenografts (systemic decorin treatment for 23 days at 10 mg/Kg) and that used for the microarray experiments, we performed qPCR as a method to validate stromal gene expression for *Mrgpra2, Gucy1a3, Siglech, Ligp, Irg1,* and *Il1b* (downregulated genes) and for *Bmp2k, Cadm1, Hey1, Peg3, Brd4, Zc3hav1* (upregulated genes). We were able to recapitulate and confirm the gene expression signatures for four (∼67%) of the downregulated genes (*Mrgpra2, Siglech, Irg1, and Il1b; P*<0.001) and for three (50%) of the upregulated genes (*Zc3hav1, Peg3,* and *Bmp2k; P*<0.001) (Figure 4). The remaining genes (*Cadm1, Hey1, Brd4, Gucy1a3,* and *Ligp*) could not be faithfully reproduced due primarily to unfavorably high thermal cycle value or lack of amplification and detection under the cycling conditions employed, suggesting low transcript number.

These data suggest a modulation of immunological genes, consistent with the aforementioned DAVID gene ontology analysis. Importantly, we demonstrate a reproducible induction of a presumed tumor suppressor gene (*Peg3*) within the stromal compartment as well as implicating a novel role for *Bmp2k* in breast cancer samples.

### Co-Cultures Mimic Decorin Regulated Stromal Genes In Vitro

**Figure 5:**
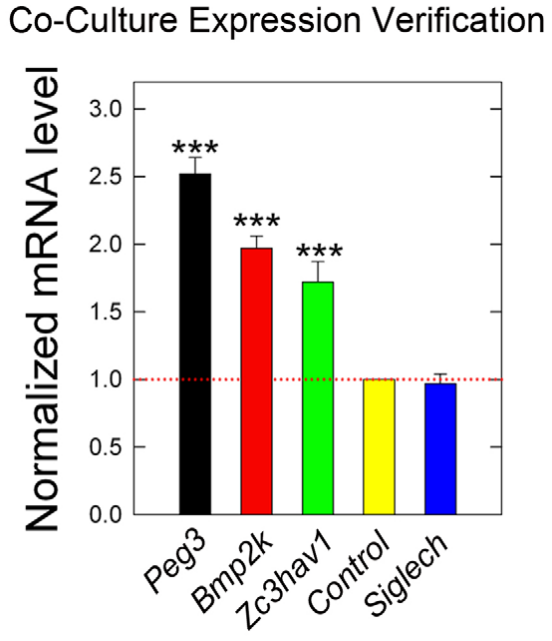
Co-Culture Gene Expression Analysis of NimbleGen Targets. Co-culture conditions were established by utilizing and plating primary mouse mammary fibroblasts followed, once at sub-confluency, by the addition of ∼10^5^ MDA-231 cells. Co-cultures were then serum starved for a 24 h period prior to exposure of 100 nM decorin protein core for a total 3–4 days under serum free conditions. Gene expression analysis was carried out via qPCR by employing the same *Mus musculus* specific primers as in the preceding section for seven of the target genes, as were reproduced in Figure 4. Recapitulation of the *in vivo* gene signature involved reproducible patterns for four of the genes (*Peg3, Bmp2K, and Zc3hav1*). Data are representative of two independent trials performed in triplicate. Fold changes reflect two independent trials employing quadruplicate replicates and analyzed with the ΔΔCt method (Please refer to Materials and Methods) and reported as the average ± SEM (***P<0.001).

We hypothesized that establishment of a co-culture approach could reconstitute in part the decorin-evoked *in vivo* stromal gene expression signatures obtained from the tumor xenografts in an *in vitro* setting. Thus, we utilized primary cell isolates of mouse mammary fibroblasts of the same genetic background (C57BL/6J) as the host for the tumor xenografts and added human mammary carcinoma MDA-231 cells to this feeder layer of primary mammary fibroblasts. Once attached, co-cultures were serum starved for 24 hours followed by chronic treatment with decorin (100 nM) for 3 days whereupon RNA was harvested and prepared as a mixed cDNA population. We focused on the expression pattern of the seven previously verified genes as reported above. Analysis of these stromal genes in context of the co-culture via qPCR revealed a recapitulation of the expression patterns of *Peg3*, *Bmp2k*, and *Zc3hav1* (Figure 5; *P*<0.001), the same stromal genes verified as induced by decorin within the tumor xenografts. However, the extents of induction among these genes were not as dramatic as shown for the verification *in vivo* (*cfr.* Figure 4). This reflects several possibilities such as the length of decorin treatment, differences in nutrient obtainment via the blood vessels and/or the lack of additional stromal cells (endothelial cells and inflammatory cells), which might synergize and ultimately prove necessary for more robust decorin-mediated induction.

It is of note to mention that expression patterns of the immunomodulatory genes could not be reconstituted since our cultures lacked immune system components and/or fibroblasts that do not normally express these genes. Finally, and although *Siglech* was highly detectable within our co-culture system, we found no significant change of its expression (Figure 5; *P*>0.05).

Collectively, these findings indicate that decorin is capable of targeting the tumor stroma in addition to the well-established anti-oncogenic activity targeting the carcinoma cells.

### Decorin Induces Peg3, Bmp2k and Zc3hav1 in a Co-culture and Tumor Xenografts

**Figure 6:**
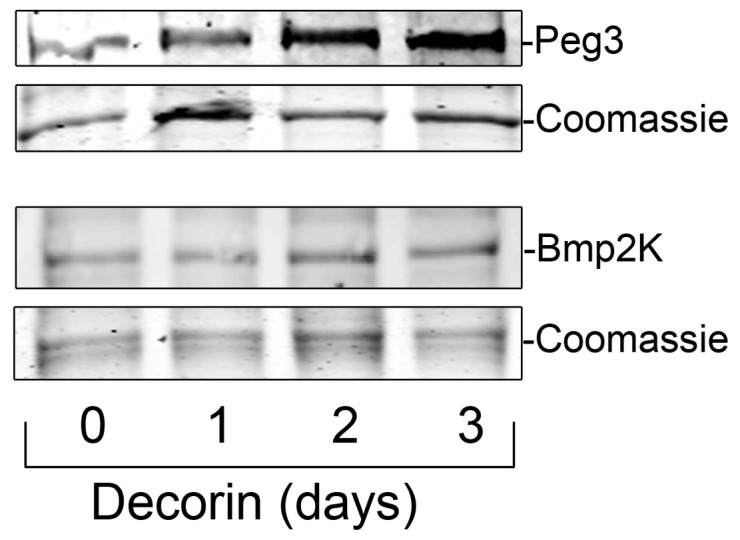
Peg3 and Bmp2 expression is induced by decorin protein core in co-cultures of MDA-231 cells and mouse mammary fibroblasts. Representative immunoblots of MDA-231 cells and mouse mammary fibroblasts in co-culture exposed to 200 nM decorin as indicated. Cell lysates were separated via SDS-PAGE and immunoblotted using an anti-Peg3 or Bmp2k primary antibodies. The bottom panels represent Coomassie Blue staining of the lower portion of the gel. The blots are representative of three independent experiments. Proteins were visualized with IR-Dye-labeled secondary antibodies and quantified using Odyssey Infrared Imaging system (LI-COR).

**Figure 7:**
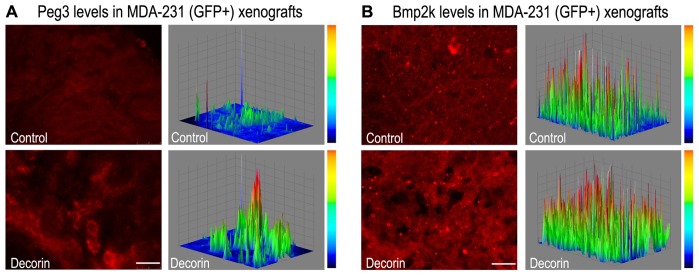
Systemic administration of decorin protein core induces Peg3 and Bmp2k levels in MDA-231(GFP+) xenografts. **A–B**: Immunofluorescence images of control and decorin-treated MDA-231(GFP+) tumor xenografts, reacted with anti-Peg3 (A) or anti-Bmp2k (B) antibodies. Mice bearing MDA-231(GFP+) tumor xenografts were treated with intraperitoneal injection of decorin protein core (10 mg/kg) every other day for 23 days. All the micrographs were taken using the same exposure and gain. Three-dimensional surface plots, on the right of each panel, were generated utilizing ImageJ software and represent Peg3 and Bmp2k expression which directly corresponds to the signal intensity obtained by the immunofluorescence. The scale bars for signal intensity are included on the right of each surface plot. Bar = 20 µm.

Following extensive qPCR analyses, we next sought to determine the levels of Peg3 and Bmp2k in a co-culture of mouse mammary fibroblasts and MDA-231 that were exposed to either vehicle (control) or decorin (200 nM) for up to 3 days. We found a marked increase of both Peg3 and Bmp2k expression in treated cells compared to controls as detected by immunoblot (Figure 6). To further strengthen the *in vivo* relevance of our data, we determined the expression of Peg3 and Bmp2k in the same orthotopic mammary tumor xenografts as used above. Systemic treatment with decorin resulted in an induction of Peg3 (Figure 7A) as well as Bmp2k (Figure 7B) and Zc3hav1 (Figure S2) within the tumor stroma as visualized via immunofluorescence and quantified using three-dimensional surface plots of the fluorescent signal. Thus, these *in vivo* data provides further proof that decorin significantly affects the tumor microenvironment via induction of Peg3, Bmp2k and Zc3hav1 at the protein and transcriptional levels as suggested by the mixed microarray data.

### Invasive Ductal Breast Carcinoma Microarray Datasets Show a Clear Reduction in PEG3

**Figure 8:**
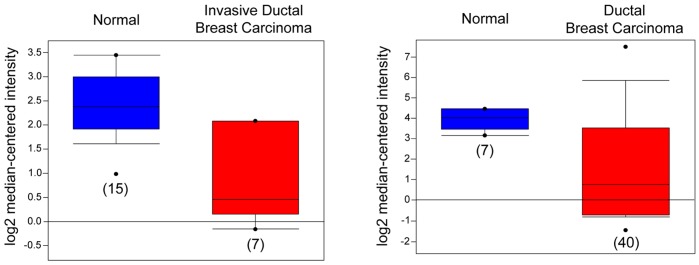
Expression array analysis of several ductal breast carcinoma microarray datasets. Statistical significance is reported as a summary statistic calculated utilizing the ONCOMINE gene expression tool [90]. As shown in the report by Karnoub *et al.* [91] (*left panel*) there was a 3.1-fold reduction of PEG3 mRNA within invasive ductal breast carcinoma (*P* = 3.64×10^−6^). Additionally, in the study by Richardson *et al*. [92] (*right panel*), PEG3 levels were found to be decreased by 5.6-fold in ductal breast carcinoma samples (*P* = 1.86×10^−6^).

Next, we analyzed *PEG3* expression in different publically available breast cancer microarray datasets using the ONCOMINE database and gene microarray data analysis tool [90]. This database was specified to query individual published microarray analyses as well as to produce a summary statistic across each distinctive gene expression study for *PEG3* mRNA levels detected in various ductal breast carcinoma samples. Two independent studies [91], [92] demonstrated a statistically significant decrease (Figure 8) of *PEG3* mRNA expression when compared to normal, non-neoplastic controls. Precisely, the dataset by Karnoub *et al.* reported a 3.1-fold decrease (*P* = 3.6×10^−6^) of *PEG3* mRNA within invasive ductal breast carcinoma (Figure 8, left panel) which is consistent with a study by Richardson *et al*, where a 5.6-fold reduction (*P* = 1.86×10^−6^) of *PEG3* expression was found in ductal breast carcinoma (Figure 8, right panel). No data were available in the ONCOMINE for either *BMP2K* or *ZC3HAV1* related to breast cancer.

These data provide further *in vivo* validation concerning the clinical relevance and potentially critical role of PEG3, functioning as a tumor suppressor, in the progression of aggressive ductal breast carcinomas. Moreover, these studies provide strong rationale for the novel induction of PEG3 within this malignancy by decorin as a possible mechanism of inhibiting tumorigenesis.

## Discussion

A current prevailing view is that tumor-associated stroma is activated by cancer cells to foster tumor growth by secreting growth factors, enhancing angiogenesis, and facilitating cell migration, ultimately culminating in metastasis to remote organ sites [93]–[97]. Thus, genes mediating tumor-stroma interactions could provide novel targets for diagnostic development and therapeutic intervention [98]. Exploratory genome-wide analysis of the tumor microenvironment in breast cancer has been limited to date. Using serial analysis of gene expression coupled with antibody-based *ex vivo* tissue fractionation, Polyak and co-workers identified 417 cell-type-specific genes among the most prominent cell types in breast cancer [97]. They demonstrated gene expression alterations in all cell types within the tumor microenvironment accompanying progression from normal breast tissue to ductal carcinoma *in situ* (DCIS) to invasive ducal carcinoma (IDC), providing evidence that these cell types all participate in tumorigenesis. Park and colleagues more recently obtained gene expression profiles of both epithelial and stromal compartments from the same tumor biopsy using laser capture microdissection (LCM) and described a stromal gene expression profile capable of predicting clinical outcome [99]. Further, using LCM, Sgroi and colleagues discovered that most of the gene expression changes take place prior to local invasion and, surprisingly, no major changes in gene expression accompanying the *in situ-*to-invasive growth transition [100].

Similarly, analysis of the tumor microenvironment by the same authors revealed, in analogy to the epithelium, that the tumor stroma undergoes extensive gene expression alterations even at the pre-invasive stage of DCIS, supporting the view that paracrine mechanisms play an important role [101]. The observed gene expression changes in the stroma associated with DCIS and IDC suggests coevolution of the tumor stroma with the tumor epithelium prior to tumor invasion.

The anti-tumorigenic properties of decorin have been presented as a stromally derived tumor repressor that functions by directly binding to receptor tyrosine kinases situated on the tumor cell membrane [73]. Recently it has been shown that this mechanism allows decorin to target potent oncoproteins such as β-catenin and Myc for degradation while presumably rendering large genetic networks that would otherwise favor continued tumorigenic growth, vulnerable to changes in gene expression signatures that would foster a less malignant state [49]. Analogously, and since decorin is a putative matrix constituent and functions as a soluble paracrine factor on the tumor proper, we sought to identify gene network changes within the broader context of the tumor microenvironment, as this compartment co-evolves with the tumor, following systemic decorin treatment on triple-negative orthotopic breast carcinoma xenograft to gain a better understanding of the molecular interplay evoked by decorin.

We found that soluble decorin protein core, acting in a paracrine capacity, is capable of substantially altering the stromal gene signature as evidenced by the differential modulation of 374 stromal genes. Gene ontology analysis revealed unexpected enrichments for suppressed genes constituting immune responses while concomitantly inducing cell adhesion and tumor suppressor genes.

Subsequently, the array yielded reproducible signatures for *Mrgpra2, Siglech, Irg1,* and *Il1b* (downregulated subset) and *Peg3, Bmp2k*, and *Zc3hav1* (induced subset). Establishment of co-cultures (primary mouse mammary fibroblasts and MDA-231 cells) allowed recapitulation of the mRNA expression pattern of the upregulated genes only; our co-culture models lacked immune-competent cells, thus the downregulated subset was not detected. Seemingly, our data presented above seem to contradict a recently published article indicating that decorin advocates for a more pro-inflammatory tumor microenvironment [85]. Only intact decorin proteoglycan, not the protein core, is able to trigger the release of TNFα or IL-12p70 from macrophages [85]. The binding of decorin protein core to TLR2/4 is not sufficient to evoke downstream signaling events, which is in contract to decorin proteoglycan. Therefore, since our present study utilized decorin protein core, the results obtained are compatible and do not contradict. Importantly, the mRNA data for *Peg3*, *Bmp2k*, and *Zc3hav1* were independently corroborated via immunofluorescence and immunoblotting in not only tumor xenografts but also in co-culture conditions, respectively, and revealed, for the first time, a novel role for *Peg3*, *Bmp2k*, and *Zc3hav1* in breast cancer progression.

Peg3 represents a unique and distinctive class among the repertoire of inherited genes by virtue of its genomically imprinted status, where in this case, the paternal allele is exclusively responsible for expressing a molecule which harbors 12 Krüppel-type zinc finger domains with two proline-rich periodic repeat domains, and is frequently rendered inactive in several malignancies [88], [89]. Consistent with a potential role as a tumor suppressor and in line with recent publications from our lab, it has been experimentally shown [102] that the N-terminal SCAN domain of Peg3 mediated direct binding to β-catenin and promotes 26S proteasomal degradation that is, surprisingly, independent of GSK3β. These studies phenocopy the pathway utilized by decorin for the non-canonical, GSK3β-independent antagonism and cessation of β-catenin signaling [40] and could provide a vital link between attenuation of RTK signaling and non-canonical β-catenin antagonism. Additionally, Peg3 has been shown to interact with and modulate the Bax/p53 axis, in concert with the TNFα and Wnt-related pathways as a mechanism to promote apoptosis [103].

Our second candidate gene is Bmp2k, a poorly understood serine/threonine kinase that plays a role in skeletal development where activation of Bmp2k is able to attenuate osteocalcin expression concomitant with reduced osteoblast differentiation [104]. Intriguingly, Bmp2k has a glutamine-rich region that shares homology to the trans-activation domain common to many transcription factors [104]. FoxC1, a transcription factor belonging to the forkhead superfamily, is subsequently able to induce the expression of Bmp2k [105]. In a separate pathological setting, a *BMP2K* variant, was reported to be strongly correlated with the development of high myopia [106].

Lastly, we were able to confirm induction of Zc3hav1 (also known as ZAP for zinc-finger antiviral protein), a gene hypothesized to encode CCCH-type zinc finger that is largely thought to prevent viral infection by retroviruses [107], particularly that triggered by HIV-1 [108]. This has novel implications on the postulated effector functions of Zc3hav1, in response to decorin, as it comprises a link to modulate innate cellular defenses against viral infections, and/or a mechanism to regulate endogenous RNA signaling within the tumor microenvironment.

Moreover, the data obtained from our co-culture suggests a potent modulatory effect on cancer-associated fibroblasts that will have a broad impact on tumor progression. Therefore, the changes in differential expression, as reported above, reflect gene signatures operative within the tumor microenvironment and thus makes assessing whether or not these changes are the result of direct effects on the tumor proper difficult. Thus, excluding the possibility of decorin protein core signaling via the fibroblast α2β1 integrin receptor, it is plausible that decorin is targeting the tumor cells directly by suppressing EGFR and Met signaling (or is integrated over several receptors), and thereby functions as a paracrine agent to elicit changes within the surrounding tumor stroma. However, this model cannot exclude decorin binding directly to receptor tyrosine kinases within the stroma (such as receptors present on endothelial cells). Importantly, we have reported that decorin severely inhibits Myc function through targeted proteasomal degradation [40] which, in turn, could have stern consequences for the tumor stroma as a favorably pro-tumorigenic environment as Myc is required for the expression of stromal genes [109]. As an alternative possibility, it would be possible through the established role of decorin in sequestering and thus indirectly inhibiting the activity of TGFβ, to elicit the immunomodulatory changes in this manner [110].

In conclusion, validation of the mixed microarray data as it pertains to stromal gene changes via several experimental methodologies performed *in vitro* and *in vivo* revealed and confirmed a novel involvement for Peg3, Bmp2k, and Zc3hav1, for the first time, in breast carcinomas where, future studies may reveal direct functions related to modulating the surrounding tumor environment as dictated by decorin. These findings provide a new paradigm for decorin protein core in controlling the tumor microenvironment as a fundamental biological mechanism with great implications for curbing tumorigenic growth by the induction of novel tumor suppressor genes within the stroma and for the discovery of novel gene signatures that could eventually help clinical assessment and prognosis.

## Materials and Methods

### Ethics Statement

All animal studies were approved by the Institutional Review Board of Thomas Jefferson University. At the end of each experiment, mice were anesthetized with isoflurane and euthanized with CO_2_ in accordance with institutional guidelines. (Approved Protocol # 196 G).

### Cells and Materials

MDA-MB-231 triple-negative breast carcinoma cells were obtained from American Type Culture Collection (Manassas, VA). MDA-231(GFP+) were previously described [111]. Cells were maintained in Dulbecco’s modified Eagle’s medium supplemented with 10% fetal bovine serum (FBS) (SAFC Biosciences, Lenexa, KS) as well as with 100 µg/ml of penicillin/streptomycin (MediaTech, Manassas, VA). Primary antibody against Peg3 (ab99252) was from Abcam Inc. (Cambridge, MA); anti-Bmp2k polyclonal antibody (PA5-11724) was from Thermo Scientific (Rockford, IL); anti-Zc3hav1 polyclonal antibody was from Abgent (San Diego, CA).

### Purification of Decorin Protein Core

Decorin protein core was purified as described elsewhere [56]. Briefly, recombinant human decorin was expressed in 293-EBNA cells as a fusion protein to poly-His_6_ within a Celligen Plus bioreactor [17]. The 293-EBNA cells were subsequently serum starved for several days in order to maximize the output of decorin in the media prior to purification. Under these conditions, the 293-EBNA cells will produce both the glycanated and unglycanated form of the recombinant decorin protein. Exploiting this method, it was possible to purify both the proteoglycan and protein core on an Ni-NTA chelating column followed by elution in increasing concentrations of imidazole (0 to 250 mM) in 20 mM Tris-HCl, 500 mM NaCl, 0.2% CHAPS, pH 8.0. Finally, the proteoglycan and core protein were separated via anion-exchange chromatography performed on Q-Sepharose and the expected doublet of decorin protein core was seen migrating with a molecular mass of ∼50 kDa [112]. Additional evidence concerning the purity of the decorin protein core preparation used was provided via an SDS-PAGE gel to analyze increasing amounts (0.5, 1, 2, and 5 µg) of decorin protein core in parallel with increasing amounts of bovine serum albumin (BSA) (2 and 5 µg). This was followed by staining the resultant gel with colloidal Coomassie blue (EZBlue™ Gel Staining Reagent, Sigma-Aldrich), which has a detection threshold of as little as 5 ng of total protein.

### Generation of Tumor Xenografts

All the animal studies performed were approved by the Institutional Review Board of Thomas Jefferson University. Twelve severe-combined immunodeficient (SCID) hairless female mice (Charles River Lab., Malvern, PA) were injected with 2×10^6^ MDA-231(GFP+) cells into the upper left mammary fat pad. Two weeks later, once tumors were established, mice were randomized into two groups. Half the mice received a daily intraperitoneal injection of recombinant protein core (10 mg/Kg). The controls received 100 µl of PBS. Three independent experiments were performed. Tumor growth was measured every day with a micro-caliper according to the following formula: V = *a*(*b* ^2^/2), where *a* and *b* represent the larger and smaller diameters of the tumor, respectively. At the end of the experiment (day 23) mice were taken to the Small Animal Imaging Facility of Thomas Jefferson University and *in vivo* expression of GFP within the xenografts was analyzed with a Kodak In-Vivo Multispectral Imaging System FX. Soon after all animals were sacrificed and all major organs were dissected and fixed in formalin, whereas tumors were cut in half and one half was snap-frozen in liquid nitrogen subsequent to RNA extraction whereas the other half was embedded in OCT compound (x) and frozen at −20°C. 10 µm thick cryostat sections were cut from the blocks and mount on superfrost slides which were then subjected to immunofluorescence staining.

### Immunofluorescence Staining of Tumor Sections

Immunofluorescence studies were performed as described before [40], [113], [114]. Frozen tissue sections of xenografts were fixed in ice cold acetone for 10 minutes. After blocking with 1% BSA, 1× PBS, sections were incubated with primary antibody against Peg3, Bmp2k or Zc3hav1 for 1 hour at room temperature. Sections were then probed with goat anti-rabbit IgG Alexa Fluor® 594 (Invitrogen). Washed sections were mounted with Prolong Gold (Invitrogen) and visualized using a Leica DM5500B microscope with Advanced Fluorescence 1.8 software (Leica Microsystems, Inc.). All the images were analyzed with Adobe Photoshop CS3 (Adobe Systems, San Jose, CA). Three-dimensional surface plots were generated utilizing ImageJ software.

### Isolation of Mouse Mammary Fibroblasts and Co-culture Experiments

Female wild type C57BL/6J mice were used to isolate primary mammary fibroblasts. Briefly, the fourth and fifth mammary glands were removed aseptically and minced with surgical blades. All the fragments were collected and diluted in 10 ml of digestion media (DMEM, 2 mg/ml collagenase type I, 50 µg/ml gentamicin) and incubated in a shaker (2 hours at 37°C) with gentle shaking. The solution was then centrifuged 10 minutes at 1000 rpm in order to eliminate floating fat cells. The obtained pellet was washed twice in 10 ml growth media (DMEM, 10% fetal bovine serum, pen/strep) containing fungizone. The cell pellet was then disaggregated by gently pipetting up and down with a 1 ml pipette tip. The cell resuspension was plated in 6-cm^2^ dishes coated with gelatin and cultured in growth media containing fungizone. After a week, the fibroblast culture was switched to growth media and passaged accordingly.

For co-culture experiments, sub-confluent primary cultures of mouse mammary fibroblasts were seeded with 1.0–1.5×10^5^ MDA-231 mammary carcinoma cells. The co-cultures were allowed to incubate for several hours for engagement of the fibroblast feeder layer. Co-cultures were then serum starved overnight whereupon chronic decorin treatment (100 nM decorin protein core) began for a total of three days. Arrival of the end-point resulted in lysing of the co-cultures in either a sufficient volume of RIPA buffer for immunoblot analyses or TRIzol (Invitrogen) to isolate total RNA and subsequent cDNA synthesis for gene expression analysis.

### Real-Time Gene Expression Verification and Analysis

Independent authentication of gene expression analysis was carried out by quantitative real-time polymerase chain reaction (qPCR) to confirm gene changes. Briefly, for *in vivo* samples, MDA-231 tumor xenografts tissue samples treated systemically with decorin protein core (10 mg/kg) for 23 days were snap-frozen with liquid nitrogen and homogenized with a mortar and pestle prior to solubilization in 2 ml of TRIzol (Invitrogen, Grand Island, NY), or alternatively, at the end point of the co-cultures (following three days of chronic 100 nM decorin protein core exposure), 6-cm^2^ dishes were lysed in 2 ml of TRIzol. Subsequently, isolated total RNA (1 µg) was annealed with oligo(dT) primers, and cDNA was synthesized utilizing the SuperScript Reverse Transcriptase II (Invitrogen) according to the manufacturer’s instructions. Gene-specific primer sets for *Mus musculus* mRNA (refer to Table S1 and Table 1) were rigorously designed and verified prior to use in qPCR. Target genes and the endogenous housekeeping gene, *Actb*, amplicons were amplified in independent reactions using Brilliant SYBR Green Master Mix II (Agilent Technologies, Cedar Creek, TX). All samples were run in triplicate with quadruplicate plate replicates on the Roche LightCycler 480-II Real Time PCR platform (Roche, Madison, Wisconsin) and the cycle number (Ct) was obtained for each independent amplicon reaction followed fold change determination via the Comparative Ct method for gene expression data analysis. Delta Ct (ΔCt) values are representative of normalized gene expression levels with respect to *Actb*. The delta delta Ct (ΔΔCt) values represent experimental cDNA (samples treated with decorin protein core) minus the corresponding ΔCt of the calibrator sample (control samples). Finally, the reported fold change represents an average of the fold changes as calculated using the double ΔCt method (2^−ΔΔCt^).

## Supporting Information

Figure S1 **SDS-PAGE gel demonstrating the purity of the decorin protein core preparation.** SDS-PAGE gel representing increasing amounts (0.5, 1, 2, and 5 µg) of decorin protein core run in parallel with increasing amounts of BSA (2 and 5 µg), as indicated. The gel was subsequently stained with colloidal Coomassie blue for highly sensitive detection (as low as 5 ng) of any co-purifying bands in the decorin protein core preparation used. The left lane indicates the migration of the molecular mass (kDa) of standard proteins.
(TIF)

Figure S2 **Systemic administration of decorin protein core** **induces Zc3hav1 levels in MDA-231(GFP+) xenografts.** **A–B**: Immunofluorescence images of control and decorin-treated MDA-231(GFP+) tumor xenografts, reacted with an anti-Zc3hav1 antibody. Mice bearing MDA-231(GFP+) tumor xenografts were treated with intraperitoneal injection of human recombinant decorin core protein (10 mg/kg) every other day for 23 days. All the micrographs were taken using the same exposure and gain. Three-dimensional surface plots, on the right of each panel, were generated utilizing ImageJ software and represent Zc3hav1 expression which directly corresponds to the signal intensity obtained by the immunofluorescence. The scale bars for signal intensity are included on the right of each surface plot. Bar = 20 µm.
(TIF)

Table S1 **Primer pairs specific for the exclusive detection of** ***Mus Musculus*** **genes with accompanying gene symbol and NCBI accession number.**
(DOCX)

## References

[1] Theocharis AD, Tzanakakis G, Karamanos NK (2010) Proteoglycans in health and disease: Novel proteoglycan roles in malignancy and their pharmacological targeting. FEBS J 277: 3904–3923. 20840587 10.1111/j.1742-4658.2010.07800.x

[2] Edwards IJ (2012) Proteoglycans in prostate cancer. Nat Rev Urology 9: 196–206. 22349653 10.1038/nrurol.2012.19

[3] Danielson KG, Fazzio A, Cohen I, Cannizzaro LA, Eichstetter I, et al (1993) The human decorin gene: intron-exon organization, discovery of two alternatively spliced exons in the 5' untranslated region, and mapping of the gene to chromosome 12q23. Genomics 15: 146–160. 8432526 10.1006/geno.1993.1022

[4] Santra M, Danielson KG, Iozzo RV (1994) Structural and functional characterization of the human decorin gene promoter. A homopurine-homopyrimidine S1 nuclease-sensitive region is involved in transcriptional control. J Biol Chem 269: 579–587. 8276854

[5] Mauviel A, Korang K, Santra M, Tewari D, Uitto J, et al (1996) Identification of a bimodal regulatory element encompassing a canonical AP-1 binding site in the proximal promoter region of the human decorin gene. J Biol Chem 271: 24824–24829. 8798756 10.1074/jbc.271.40.24824

[6] Adany R, Iozzo RV (1990) Altered methylation of versican proteoglycan gene in human colon carcinoma. Biochem Biophys Res Commun 171: 1402–1413. 2222452 10.1016/0006-291x(90)90841-a

[7] Adany R, Iozzo RV (1991) Hypomethylation of the decorin proteoglycan gene in human colon cancer. Biochem J 276: 301–306. 1710888 10.1042/bj2760301 PMC1151091

[8] Coppock DL, Kopman C, Scandalis S, Gilleran S (1993) Preferential gene expression in quiescent human lung fibroblasts. Cell Growth & Differ 4: 483–493. 8396966

[9] Mauviel A, Santra M, Chen YQ, Uitto J, Iozzo RV (1995) Transcriptional regulation of decorin gene expression. Induction by quiescence and repression by tumor necrosis factor-α. J Biol Chem 270: 11692–11700. 7744809 10.1074/jbc.270.19.11692

[10] Kähäri V-M, Larjava H, Uitto J (1991) Differential regulation of extracellular matrix proteoglycan (PG) gene expression. Transforming growth factor-β-1 up-regulates biglycan (PGI), and versican (large fibroblast PG) but down- regulates decorin (PGII) mRNA levels in human fiborblasts in culture. J Biol Chem 266: 10608–10615. 2037600

[11] Kolettas E, Rosenberger RF (1998) Suppression of decorin expression and partial induction of anchorage-independent growth by the *v-src* oncogene in human fibroblasts. Eur J Biochem 254: 266–274. 9660179 10.1046/j.1432-1327.1998.2540266.x

[12] Iozzo RV (1988) Proteoglycans and neoplasia. Cancer Metastasis Rev 7: 39–50. 3293831 10.1007/BF00048277

[13] Iozzo RV (1998) Matrix proteoglycans: from molecular design to cellular function. Annu Rev Biochem 67: 609–652. 9759499 10.1146/annurev.biochem.67.1.609

[14] Iozzo RV (1999) The biology of the small leucine-rich proteoglycans. Functional network of interactive proteins. J Biol Chem 274: 18843–18846. 10383378 10.1074/jbc.274.27.18843

[15] Keene DR, San Antonio JD, Mayne R, McQuillan DJ, Sarris G, et al (2000) Decorin binds near the C terminus of type I collagen. J Biol Chem 275: 21801–21804. 10823816 10.1074/jbc.C000278200

[16] Häkkinen L, Strassburger S, Kahari VM, Scott PG, Eichstetter I, et al (2000) A role for decorin in the structural organization of periodontal ligament. Lab Invest 80: 1869–1880. 11140699 10.1038/labinvest.3780197

[17] Goldoni S, Owens RT, McQuillan DJ, Shriver Z, Sasisekharan R, et al (2004) Biologically active decorin is a monomer in solution. J Biol Chem 279: 6606–6612. 14660661 10.1074/jbc.M310342200

[18] Schaefer L, Iozzo RV (2008) Biological functions of the small leucine-rich proteoglycans: from genetics to signal transduction. J Biol Chem 283: 21305–21309. 18463092 10.1074/jbc.R800020200 PMC2490788

[19] Zhang G, Ezura Y, Chervoneva I, Robinson PS, Beason DP, et al (2006) Decorin regulates assembly of collagen fibrils and acquisition of biomechanical properties during tendon development. J Cell Biochem 98: 1436–1449. 16518859 10.1002/jcb.20776

[20] Robinson PS, Lin TW, Jawad AF, Iozzo RV, Soslowsky LJ (2004) Investigating tendon fascicle structure-function relationship in a transgenic age mouse model using multiple regression models. Ann Biomed Eng 32: 924–931. 15298430 10.1023/b:abme.0000032455.78459.56

[21] Robinson PS, Huang TF, Kazam E, Iozzo RV, Birk DE, et al (2005) Influence of decorin and biglycan on mechanical properties of multiple tendons in knockout mice. J Biomechanical Eng 127: 181–185. 10.1115/1.1835363 15868800

[22] Iozzo RV, Goldoni S, Berendsen A, Young MF (2011) Small leucine-rich proteoglycans. In: Mecham RP, editors. Extracellular Matrix: An overview. Springer. 197–231.

[23] Kalamajski S, Oldberd Å (2010) The role of small leucine-rich proteoglycans in collagen fibrillogenesis. Matrix Biol 29: 248–253. 20080181 10.1016/j.matbio.2010.01.001

[24] Hildebrand A, Romaris M, Rasmussen LM, Heinegård D, Twardzik DR, et al (1994) Interaction of the small interstitial proteoglycans biglycan, decorin and fibromodulin with transforming growth factor β. Biochem J 302: 527–534. 8093006 10.1042/bj3020527 PMC1137259

[25] Ferdous Z, Wei VM, Iozzo RV, Höök M, Grande-Allen KJ (2007) Decorin-transforming growth factor-β interaction regulates matrix organization and mechanical characteristics of three-dimensional collagen matrices. J Biol Chem 282: 35887–35898. 17942398 10.1074/jbc.M705180200

[26] Baghy K, Dezsó K, László V, Fullár A, Péterfia B, et al (2011) Ablation of the decorin gene enhances experimental hepatic fibrosis and impairs hepatic healing in mice. Lab Invest 91: 439–451. 20956977 10.1038/labinvest.2010.172 PMC5074558

[27] Baghy K, Iozzo RV, Kovalszky I (2012) Decorin-TGFβ axis in hepatic fibrosis and cirrhosis. J Histochem Cytochem 60: 262–268. 22260996 10.1369/0022155412438104 PMC3351239

[28] Brown EL, Wooten RM, Johnson BJ, Iozzo RV, Smith A, et al (2001) Resistance to Lyme disease in decorin-deficient mice. J Clin Invest 107: 845–852. 11285303 10.1172/JCI11692 PMC199574

[29] Liang FT, Wang T, Brown EL, Iozzo RV, Fikrig E (2004) Protective niche for *Borrelia burgdorferi* to evade humoral immunity. Am J Pathol 165: 977–985. 15331421 10.1016/S0002-9440(10)63359-7 PMC1618599

[30] Iozzo RV, Schaefer L (2010) Proteoglycans in health and disease: Novel regulatory signaling mechanisms evoked by the small leucine-rich proteoglycans. FEBS J 277: 3864–3875. 20840584 10.1111/j.1742-4658.2010.07797.x PMC3000440

[31] Zhu J-X, Goldoni S, Bix G, Owens RA, McQuillan D, et al (2005) Decorin evokes protracted internalization and degradation of the EGF receptor via caveolar endocytosis. J Biol Chem 280: 32468–32479. 15994311 10.1074/jbc.M503833200

[32] Reed CC, Gauldie J, Iozzo RV (2002) Suppression of tumorigenicity by adenovirus-mediated gene transfer of decorin. Oncogene 21: 3688–3695. 12032837 10.1038/sj.onc.1205470

[33] Csordás G, Santra M, Reed CC, Eichstetter I, McQuillan DJ, et al (2000) Sustained down-regulation of the epidermal growth factor receptor by decorin. A mechanism for controlling tumor growth *in vivo* . J Biol Chem 275: 32879–32887. 10913155 10.1074/jbc.M005609200

[34] Santra M, Eichstetter I, Iozzo RV (2000) An anti-oncogenic role for decorin: downregulation of ErbB2 leads to growth suppression and cytodifferentiation of mammary carcinoma cells. J Biol Chem 275: 35153–35161. 10942781 10.1074/jbc.M006821200

[35] Schönherr E, Sunderkötter C, Iozzo RV, Schaefer L (2005) Decorin, a novel player in the insulin-like growth factor system. J Biol Chem 280: 15767–15772. 15701628 10.1074/jbc.M500451200

[36] Schaefer L, Tsalastra W, Babelova A, Baliova M, Minnerup J, et al (2007) Decorin-mediated regulation of fibrillin-1 in the kidney involves the insulin-like growth factor-1 receptor and mammalian target of rapamycin. Am J Pathol 170: 301–315. 17200203 10.2353/ajpath.2007.060497 PMC1762680

[37] Merline R, Lazaroski S, Babelova A, Tsalastra-Greul W, Pfeilschifter J, et al (2009) Decorin deficiency in diabetic mice: aggravation of nephropathy due to overexpression of profibrotic factors, enhanced apoptosis and mononuclear cell infiltration. J Physiol Pharmacol 60 suppl 4 5–13. PMC6714047 20083846

[38] Iozzo RV, Buraschi S, Genua M, Xu S-Q, Solomides CC, et al (2011) Decorin antagonizes IGF receptor I (IGF-IR) function by interfering with IGF-IR activity and attenuating downstream signaling. J Biol Chem 286: 34712–34721. 21840990 10.1074/jbc.M111.262766 PMC3186372

[39] Goldoni S, Humphries A, Nyström A, Sattar S, Owens RT, et al (2009) Decorin is a novel antagonistic ligand of the Met receptor. J Cell Biol 185: 743–754. 19433454 10.1083/jcb.200901129 PMC2711571

[40] Buraschi S, Pal N, Tyler-Rubinstein N, Owens RT, Neill T, et al (2010) Decorin antagonizes Met receptor activity and downregulates β-catenin and Myc levels. J Biol Chem 285: 42075–42085. 20974860 10.1074/jbc.M110.172841 PMC3009933

[41] Schaefer L, Iozzo RV (2012) Small leucine-rich proteoglycans, at the crossroad of cancer growth and inflammation. Curr Opin Genet Dev 22: 56–57. 22326829 10.1016/j.gde.2011.12.002

[42] Seidler DG, Goldoni S, Agnew C, Cardi C, Thakur ML, et al (2006) Decorin protein core inhibits *in vivo* cancer growth and metabolism by hindering epidermal growth factor receptor function and triggering apoptosis via caspase-3 activation. J Biol Chem 281: 26408–26418. 16835231 10.1074/jbc.M602853200

[43] Patel S, Santra M, McQuillan DJ, Iozzo RV, Thomas AP (1998) Decorin activates the epidermal growth factor receptor and elevates cytosolic Ca^2+^ in A431 cells. J Biol Chem 273: 3121–3124. 9452417 10.1074/jbc.273.6.3121

[44] De Luca A, Santra M, Baldi A, Giordano A, Iozzo RV (1996) Decorin-induced growth suppression is associated with upregulation of p21, an inhibitor of cyclin-dependent kinases. J Biol Chem 271: 18961–18965. 8702560 10.1074/jbc.271.31.18961

[45] Santra M, Mann DM, Mercer EW, Skorski T, Calabretta B, et al (1997) Ectopic expression of decorin protein core causes a generalized growth suppression in neoplastic cells of various histogenetic origin and requires endogenous p21, an inhibitor of cyclin-dependent kinases. J Clin Invest 100: 149–157. 9202067 10.1172/JCI119507 PMC508175

[46] Moore D, Kohn AD, De Ferrari GV, Kaykas A (2004) Wnt and β-catenin signaling: Diseases and therapies. Nat Rev Genet 5: 689–699. 10.1038/nrg1427 15372092

[47] Albihn A, Johnsen JI, Henriksson MA (2010) MYC in oncogenesis and as a target for cancer therapies. Adv Cancer Res 107: 163–224. 20399964 10.1016/S0065-230X(10)07006-5

[48] Jung P, Menssen A, Mayr D, Hermeking H (2008) AP4 encodes a c-Myc-inducible repressor of *p21* . Proc Natl Acad Sci USA 105: 15046–15051. 18818310 10.1073/pnas.0801773105 PMC2553359

[49] Neill T, Schaefer L, Iozzo RV (2012) Decorin, a guardian from the matrix. Am J Pathol 181: 380–387. 22735579 10.1016/j.ajpath.2012.04.029 PMC3409438

[50] Bi X, Tong C, Dokendorff A, Banroft L, Gallagher L, et al (2008) Genetic deficiency of decorin causes intestinal tumor formation through disruption of intestinal cell maturation. Carcinogenesis 29: 1435–1440. 18550571 10.1093/carcin/bgn141 PMC2811538

[51] Iozzo RV, Chakrani F, Perrotti D, McQuillan DJ, Skorski T, et al (1999) Cooperative action of germline mutations in decorin and p53 accelerates lymphoma tumorigenesis. Proc Natl Acad Sci USA 96: 3092–3097. 10077642 10.1073/pnas.96.6.3092 PMC15900

[52] Schönherr E, Sunderkotter C, Schaefer L, Thanos S, Grässel S, et al (2004) Decorin deficiency leads to impaired angiogenesis in injured mouse cornea. J Vasc Res 41: 499–508. 15528932 10.1159/000081806

[53] Järveläinen H, Puolakkainen P, Pakkanen S, Brown EL, Höök M, et al (2006) A role for decorin in cutaneous wound healing and angiogenesis. Wound Rep Reg 14: 443–452. 10.1111/j.1743-6109.2006.00150.x 16939572

[54] Grant DS, Yenisey C, Rose RW, Tootell M, Santra M, et al (2002) Decorin suppresses tumor cell-mediated angiogenesis. Oncogene 21: 4765–4777. 12101415 10.1038/sj.onc.1205595

[55] Neill T, Painter H, Buraschi S, Owens RT, Lisanti MP, et al (2012) Decorin antagonizes the angiogenic network. Concurrent inhibition of Met, hypoxia inducible factor-1α and vascular endothelial growth factor A and induction of thrombospondin-1 and TIMP3. J Biol Chem 287: 5492–5506. 22194599 10.1074/jbc.M111.283499 PMC3285326

[56] Reed CC, Waterhouse A, Kirby S, Kay P, Owens RA, et al (2005) Decorin prevents metastatic spreading of breast cancer. Oncogene 24: 1104–1110. 15690056 10.1038/sj.onc.1208329

[57] Goldoni S, Seidler DG, Heath J, Fassan M, Baffa R, et al (2008) An anti-metastatic role for decorin in breast cancer. Am J Pathol 173: 844–855. 18688028 10.2353/ajpath.2008.080275 PMC2527080

[58] Ständer M, Naumann U, Dumitrescu L, Heneka M, Löschmann P, et al (1998) Decorin gene transfer-mediated suppression of TGF-β synthesis abrogates experimental malignant glioma growth in vivo. Gene Therapy 5: 1187–1194. 9930319 10.1038/sj.gt.3300709

[59] Tralhão JG, Schaefer L, Micegova M, Evaristo C, Schönherr E, et al (2003) In vivo selective and distant killing of cancer cells using adenovirus-mediated decorin gene transfer. FASEB J 17: 464–466. 12631584 10.1096/fj.02-0534fje PMC5913819

[60] Biglari A, Bataille D, Naumann U, Weller M, Zirger J, et al (2004) Effects of ectopic decorin in modulating intracranial glioma progression *in vivo*, in a rat syngeneic model. Cancer Gene Therapy 11: 721–732. 15475879 10.1038/sj.cgt.7700783 PMC2902255

[61] Araki K, Wakabayashi H, Shintani K, Morikawa J, Matsumine A, et al (2009) Decorin suppresses bone metastasis in a breast cancer cell line. Oncology 77: 92–99. 19590249 10.1159/000228253

[62] Hu Y, Sun H, Owens RT, Wu J, Chen YQ, et al (2009) Decorin suppresses prostate tumor growth through inhibition of epidermal growth factor and androgen receptor pathways. Neoplasia 11: 1042–1053. 19794963 10.1593/neo.09760 PMC2745670

[63] Leygue E, Snell L, Dotzlaw H, Troup S, Hiller-Hitchcock T, et al (2000) Lumican and decorin are differentially expressed in human breast carcinoma. J Pathol 192: 313–320. 11054714 10.1002/1096-9896(200011)192:3<313::AID-PATH694>3.0.CO;2-B

[64] Troup S, Njue C, Kliewer EV, Parisien M, Roskelley C, et al (2003) Reduced expression of the small leucine-rich proteoglycans, lumican, and decorin is associated with poor outcome in node-negative invasive breast cancer. Clin Cancer Res 9: 207–214. 12538471

[65] Biaoxue R, Xiguang C, Hua L, Hui M, Shuanying Y, et al (2011) Decreased expression of decorin and p57 (KIP2) correlates with poor survival and lymphatic metastasis in lung cancer patients. Int J Biol Markers 26: 9–21. 21360479 10.5301/jbm.2011.6372

[66] Alowami S, Troup S, Al-Haddad S, Kirkpatrick I, Watson PH (2003) Mammographic density is related to stroma and stromal proteoglycan expression. Breast Cancer Res 5: R129–R135. 12927043 10.1186/bcr622 PMC314426

[67] Provenzano PP, Inman DR, Eliceiri KW, Knittel JG, Yan L, et al (2008) Collagen density promotes mammary tumor initiation and progression. BMC Medicine 6: 11. 18442412 10.1186/1741-7015-6-11 PMC2386807

[68] Maller O, Martinson H, Schedin P (2010) Extracellular matrix composition reveals complex and dynamic stromal-epithelial interactions in the mammary gland. J Mammary Gland Biol Neoplasia 15: 301–318. 20811805 10.1007/s10911-010-9189-6

[69] Howell A, Landberg G, Bergh J (2009) Breast tumor stroma is a prognostic indicator and a target for therapy. Breast Cancer Res (Suppl 3): S16. 10.1186/bcr2435 PMC2797696 20030867

[70] Schedin P, O'Brien J, Rudolph M, Stein T, Borges V (2007) Microenvironment of the involuting mammary gland mediates mammary cancer progression. J Mammary Gland Biol Neoplasia 12: 71–82. 17318269 10.1007/s10911-007-9039-3

[71] Iozzo RV, Cohen I (1993) Altered proteoglycan gene expression and the tumor stroma. Cell Mol Life Sci 49: 447–455. 10.1007/BF01923588 8500599

[72] Iozzo RV (1995) Tumor stroma as a regulator of neoplastic behavior. Agonistic and antagonistic elements embedded in the same connective tissue. Lab Invest 73: 157–160. 7637316

[73] Goldoni S, Iozzo RV (2008) Tumor microenvironment: Modulation by decorin and related molecules harboring leucine-rich tandem motifs. Int J Cancer 123: 2473–2479. 18798267 10.1002/ijc.23930

[74] Huang DW, Sherman BT, Lempicki RA (2009) Systematic and integrative analysis of large gene lists using DAVID bioinformatics resources. Nat Prot 4: 44–57. 10.1038/nprot.2008.211 19131956

[75] Chen B, Zhang D, Pollard JW (2003) Progesterone regulation of the mammalian ortholog of methylcitrate dehydratase (*Immune Response Gene 1*) in uterine epithelium during implantation through the protein kinase C pathway. Mol Endocrinol 17: 2340–2354. 12893884 10.1210/me.2003-0207

[76] Lee CGL, Jenkins NA, Gilbert DJ, Copeland NG, O'Briend WE (1995) Cloning and analysis of gene regulation of a novel LPS-inducible cDNA. Immunogenetics 41: 263–270. 7721348 10.1007/BF00172150

[77] Degrandi D, Hoffmann R, Beuter-Gunia C, Pfeffer K (2009) The proinflammatory cytokine-induced IRG1 protein associates with mitochondria. J Interferon Cytokine Res 29: 55–67. 19014335 10.1089/jir.2008.0013

[78] von Gunten S, Bochner BS (2008) Basic and clinical immunology of Siglecs. Ann N Y Acad Sci 1143: 61–68. 19076345 10.1196/annals.1443.011 PMC3902170

[79] Zerrahn J, Schaible UE, Brinkmann V, Guhlich U, Kaufman SHE (2002) The IFN-Inducible Golgi- and endoplasmic reticulum-associated 47-kDa GTPase IIGP is transiently expressed during listeriosis. J Immunol 168: 3428–3436. 11907101 10.4049/jimmunol.168.7.3428

[80] Sugerman PB, Faber SB, Willis LM, Petrovic A, Murphy GF, et al (2004) Kinetics of gene expression in murine cutaneous graft-*versus*-host disease. Am J Pathol 164: 2189–2202. 15161652 10.1016/S0002-9440(10)63776-5 PMC1615752

[81] Dharajiya N, Vaidya S, Sinha M, Luxon B, Boldogh I, et al (2009) Allergen challange induces Ifng dependent GTPases in the lungs as part of Th1 transcriptome response in a murine model of allergic asthma. PLoS ONE 4: e8172. 20027288 10.1371/journal.pone.0008172 PMC2791840

[82] Vesely MD, Kershaw MH, Schreiber RD, Smyth MJ (2011) Natural innate and adaptive immunity to cancer. Ann Rev Immunol 29: 235–271. 21219185 10.1146/annurev-immunol-031210-101324

[83] Marchica CL, Pinelli V, Borges M, Zummer J, Narayanan V, et al (2011) A role for decorin in a murine model of allergen-induced asthma. Am J Physiol Lung Cell Mol Physiol 300: 863–873. 10.1152/ajplung.00300.2009 PMC5243205 21378022

[84] Seidler DG, Mohamed NA, Bocian C, Stadtmann A, Hermann S, et al (2011) The role for decorin in delayed-type hypersensitivity. J Immunol 187: 6108–6199. 22043007 10.4049/jimmunol.1100373 PMC5070385

[85] Merline R, Moreth K, Beckmann J, Nastase MV, Zeng-Brouwers J, et al (2011) Signaling by the matrix proteoglycan decorin controls inflammation and cancer through PDCD4 and microRNA-21. Sci Signal 4: ra75. 22087031 10.1126/scisignal.2001868 PMC5029092

[86] Liang Q-L, Chen G-Q, Li Z-Y, Wang B-R (2011) Function and histopathology of a cell adhesion molecule TSLC1 in cancer. Cancer Invest 29: 107–112. 21329006 10.3109/07357907.2010.543211

[87] Heller G, Geradts J, Ziegler B, Newsham I, Filipitis M, et al (2007) Downregulation of TSLC1 and DAL-1 expression occurs frequently in breast cancer. Breast Cancer Res Treat 103: 283–291. 17260099 10.1007/s10549-006-9377-7

[88] Feng W, Marquez RT, Lu Z, Liu J, Lu KH, et al (2008) Imprinted tumor suppressor genes *ARHI* and *PEG3* are the most frequently down-regulated in human ovarian cancers by loss of heterozygosity and promoter methylation. Cancer 112: 1489–1502. 18286529 10.1002/cncr.23323

[89] Maegawa S, Yoshioka H, Itaba N, Kubota N, Nishihara S, et al (2001) Epigenetic silencing of PEG3 gene expression in human glioma cell lines. Mol Carcinogenesis 31: 1–9. 10.1002/mc.1034 11398192

[90] Rhodes DR, Yu J, Shanker K, Deshpande N, Varambally R, et al (2004) Large-scale meta-analysis of cancer microarray data identifies common transcriptional profiles of neoplastic transformation and progression. Proc Natl Acad Sci USA 101: 9309–9314. 15184677 10.1073/pnas.0401994101 PMC438973

[91] Karnoub AE, Dash AB, Vo AP, Sullivan A, Brooks MW, et al (2007) Mesenchymal stem cells within tumor stroma promote breast cancer metastasis. Nature 449: 557–565. 17914389 10.1038/nature06188

[92] Richardson AL, Wang ZC, De Nicolo A, Lu X, Brown M, et al (2006) X chromosomal abnormalities in basal-like human breast cancer. Cancer Cell 9: 121–132. 16473279 10.1016/j.ccr.2006.01.013

[93] Bissell MJ, Radisky D (2001) Putting tumors in context. Nat Rev Cancer 1: 46–54. 11900251 10.1038/35094059 PMC2975572

[94] Rønnov-Jessen L, Bissell MJ (2009) Breast cancer by proxy: can the microenvironment be both the cause and consequence? Trends Mol Med 15: 5–13. 19091631 10.1016/j.molmed.2008.11.001 PMC2746030

[95] Orimo A, Gupta PB, Sgroi DC, Arenzana-Seisdedos F, Delaunay T, et al (2005) Stromal fibroblasts present in invasive human breast carcinomas promote tumor growth and angiogenesis through elevated SDF-1/CXCL12 secretion. Cell 121: 335–348. 15882617 10.1016/j.cell.2005.02.034

[96] Nikitovic D, Berdiaki K, Chalkiadaki G, Karamanos N, Tzanakakis G (2008) The role of SLRP-Proteoglycans in osteosarcoma pathogenesis. Connective Tissue Res 49: 235–238. 10.1080/03008200802147589 18661350

[97] Allinen M, Beroukhim R, Cai L, Brennan C, Lahti-Domenici J, et al (2004) Molecular characterization of the tumor microenvironment in breast cancer. Cancer Cell 6: 17–32. 15261139 10.1016/j.ccr.2004.06.010

[98] Iozzo RV, Sanderson RD (2011) Proteoglycans in cancer biology, tumour microenvironment and angiogenesis. J Cell Mol Med 15: 1013–1031. 21155971 10.1111/j.1582-4934.2010.01236.x PMC3633488

[99] Finak G, Bertos N, Pepin F, Sadekova S, Souleimanova M, et al (2008) Stromal gene expression predicts clinical outcome in breast cancer. Nat Med 14: 518–527. 18438415 10.1038/nm1764

[100] Ma X-J, Salunga R, Tuggle JT, Gaudet J, Enright E, et al (2003) Gene expression profiles of human breast cancer progression. Proc Natl Acad Sci USA 100: 5974–5979. 12714683 10.1073/pnas.0931261100 PMC156311

[101] Ma X-J, Dahiya S, Richardson E, Erlander M, Sgroi DC (2009) Gene expression profiling of the tumor microenvironment during breast cancer progression. Breast Cancer Res 11: R7. 19187537 10.1186/bcr2222 PMC2687710

[102] Jiang X, Yu Y, Yang HW, Agar NYR, Frado L, et al (2010) The imprinted gene *PEG3* inhibits Wnt signaling and regulates glioma growth. J Biol Chem 285: 8472–8480. 20064927 10.1074/jbc.M109.069450 PMC2832996

[103] Deng Y, Wu X (2000) Peg3/Pw1 promotes p53-mediated apoptosis by inducing Bax translocation from cytosol to mitochondria. Proc Natl Acad Sci USA 97: 12050–12055. 11050235 10.1073/pnas.97.22.12050 PMC17292

[104] Kearns AE, Donohue MM, Sanyal B, Demay MB (2001) Cloning and characterization of a novel protein kinase that impairs osteoblast differentiation *in vitro* . J Biol Chem 276: 42213–42218. 11500515 10.1074/jbc.M106163200

[105] Tamimi Y, Lines M, Coca-Prados M, Walter MA (2004) Identification of target genes regulated by *FOXC1* using nickel agarose-based chromatin enrichment. Invest Ophthalmol Vis Sci 45: 3904–3913. 15505035 10.1167/iovs.04-0628

[106] Liu H-P, Lin Y-J, Lin W-YWL, Sheu JJ-C, Lin H-J, et al (2009) A novel genetic variant of *BMP2K* contributes to high myopia. J Clin Lab Anal 23: 362–367. 19927351 10.1002/jcla.20344 PMC6649202

[107] Gao G, Guo X, Goff SP (2002) Inhibition of retroviral RNA production by ZAP, a CCCH-type zinc finger protein. Science 297: 1703–1706. 12215647 10.1126/science.1074276

[108] Zhu Y, Chen G, Lv F, Wang X, Ji X, et al (2011) Zinc-finger antiviral protein inhibits HIV-1 infection by selectively targeting multiply spliced viral mRNAs for degradation. Proc Natl Acad Sci USA 108: 15834–15839. 21876179 10.1073/pnas.1101676108 PMC3179061

[109] Sodir NM, Swigart LB, Karnezis AN, Hanahan D, Evan GI, et al (2011) Endogenous Myc maintains the tumor microenvironment. Genes Dev 25: 907–916. 21478273 10.1101/gad.2038411 PMC3084025

[110] Salnikov AV, Roswall P, Sundberg C, Gardner H, Heldin N-E, et al (2005) Inhibition of TGF-β modulates macrophages and vessel maturation in parallel to a lowering of interstitial fluid pressure in experimental carcinoma. Lab Invest 85: 512–521. 15711566 10.1038/labinvest.3700252

[111] Chiavarina B, Whitaker-Menezes D, Migneco G, Martinez-Outschoorn UE, Pavlides S, et al (2010) HIF1-alpha functions as a tumor promoter in cancer associated fibroblasts, and as a tumor suppressor in breast cancer cells. Autophagy drives compartment-specific oncogenesis. Cell Cycle 9: 3534–3551. 20864819 10.4161/cc.9.17.12908 PMC3047618

[112] Ramamurthy P, Hocking AM, McQuillan DJ (1996) Recombinant decorin glycoforms. Purification and structure. J Biol Chem 271: 19578–19584. 8702652 10.1074/jbc.271.32.19578

[113] Cohen IR, Murdoch AD, Naso MF, Marchetti D, Berd D, et al (1994) Abnormal expression of perlecan proteoglycan in metastatic melanomas. Cancer Res 54: 5771–5774. 7954396

[114] Ryynänen M, Ryynänen J, Solberg S, Iozzo RV, Knowlton RG, et al (1992) Genetic linkage of Type VII collagen (COL7A1) to dominant dystrophic epidermolysis bullosa in families with abnormal anchoring fibrils. J Clin Invest 89: 974–980. 1347297 10.1172/JCI115680 PMC442946

