# Supplementary figures and images for: Decorin Protein Core Affects the Global Gene Expression Profile of the Tumor Microenvironment in a Triple-Negative Orthotopic Breast Carcinoma Xenograft Model
Source: PLoS One. 2012 Sep 19;7(9):e45559. doi: 10.1371/journal.pone.0045559 (PMC3446891; doi:10.1371/journal.pone.0045559)

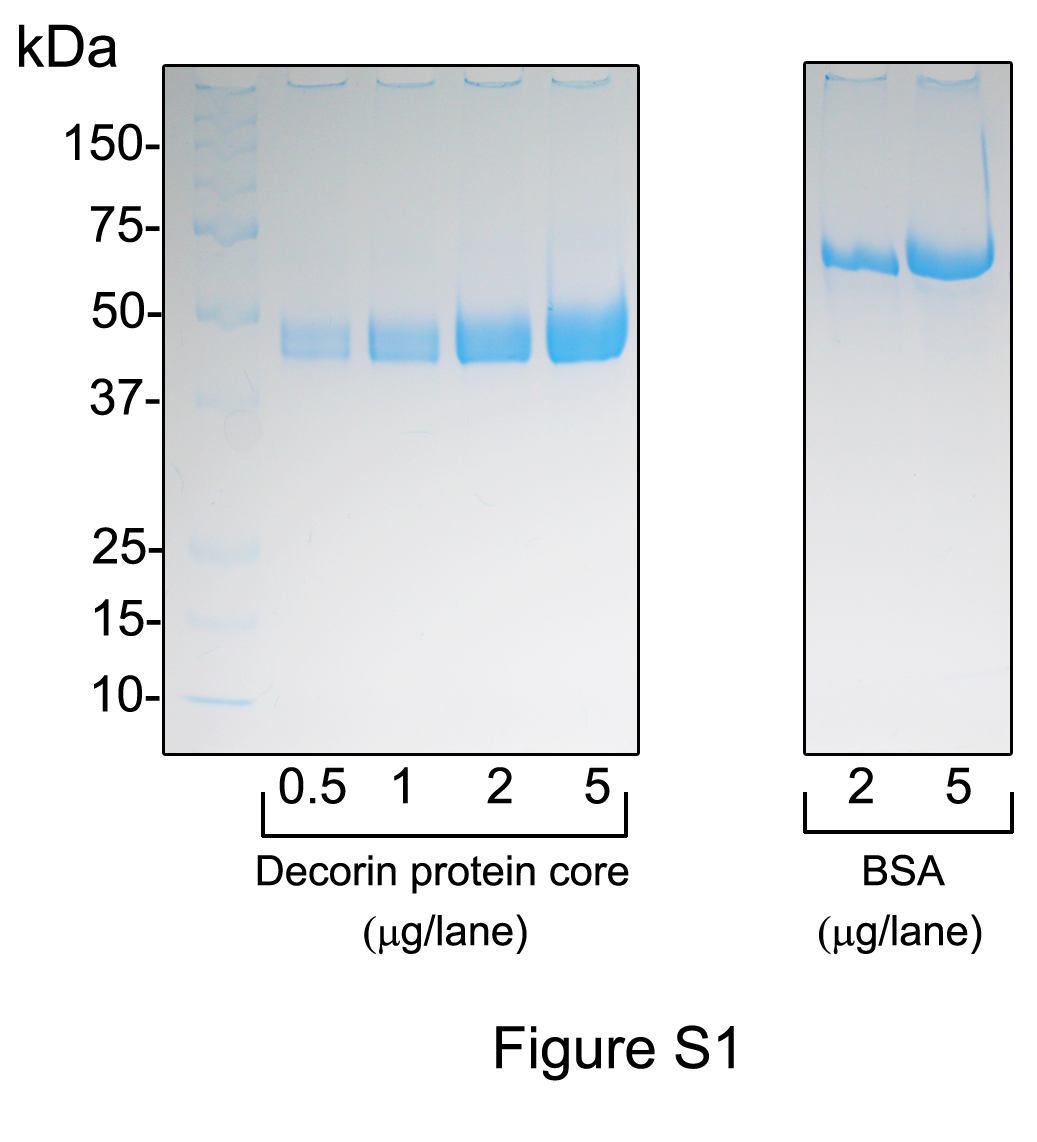

Supplement: Figure S1 — SDS-PAGE gel demonstrating the purity of the decorin protein core preparation. SDS-PAGE gel representing increasing amounts (0.5, 1, 2, and 5 µg) of decorin protein core run in parallel with increasing amounts of BSA (2 and 5 µg), as indicated. The gel was subsequently stained with colloidal Coomassie blue for highly sensitive detection (as low as 5 ng) of any co-purifying bands in the decorin protein core preparation used. The left lane indicates the migration of the molecular mass (kDa) of standard proteins. (TIF) [file pone.0045559.s001.tif]

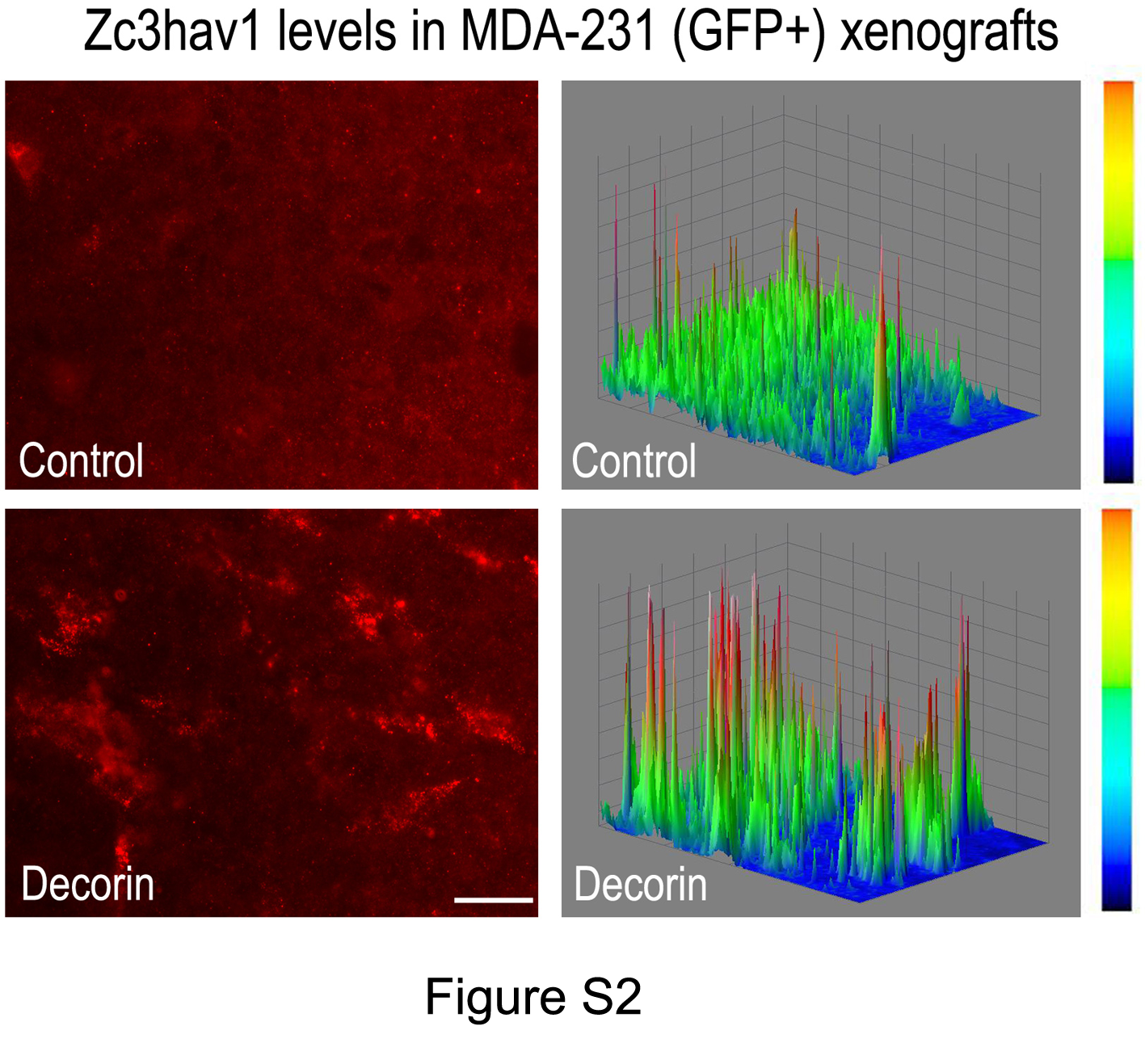

Supplement: Figure S2 — Systemic administration of decorin protein core induces Zc3hav1 levels in MDA-231(GFP+) xenografts. A–B: Immunofluorescence images of control and decorin-treated MDA-231(GFP+) tumor xenografts, reacted with an anti-Zc3hav1 antibody. Mice bearing MDA-231(GFP+) tumor xenografts were treated with intraperitoneal injection of human recombinant decorin core protein (10 mg/kg) every other day for 23 days. All the micrographs were taken using the same exposure and gain. Three-dimensional surface plots, on the right of each panel, were generated utilizing ImageJ software and represent Zc3hav1 expression which directly corresponds to the signal intensity obtained by the immunofluorescence. The scale bars for signal intensity are included on the right of each surface plot. Bar = 20 µm. (TIF) [file pone.0045559.s002.tif]
