# Supplementary material for: Decorin Protein Core Affects the Global Gene Expression Profile of the Tumor Microenvironment in a Triple-Negative Orthotopic Breast Carcinoma Xenograft Model
Source: PLoS One. 2012 Sep 19;7(9):e45559. doi: 10.1371/journal.pone.0045559 (PMC3446891; doi:10.1371/journal.pone.0045559)
Supplement: Table S1 — Primer pairs specific for the exclusive detection of Mus Musculus genes with accompanying gene symbol and NCBI accession number. (DOCX) [file pone.0045559.s003.docx]

| **Official Gene Symbol** | **NCBI**  **Accession Number** | ***Mus Musculus* Specific Forward Primer**  **(5’ to 3’)** | ***Mus Musculus* Specific Reverse Primer**  **(5’ to 3’)** |
| --- | --- | --- | --- |
| *Bmp2k* | NM_080708.1 | 5’-GCAACTTCATTTGCATCGTCATCC-3’ | 5’-GCCCAGGTCAAAGGTTTGGAAGC-3’ |
| *Mrgpra2* | NM_153101.3 | 5’-aagcatgctcagtgccatc -3’ | 5’-gataccaggtggggcaca-3’ |
| *Cadm1* | NM_207675.2 | 5’-gtctcaatctcggatgaaggg-3’ | 5’-tgccgtgtctttctggatatc-3’ |
| *Hey1* | NM_010423.2 | 5’-CGCTCCGCCACCATGAAGAG-3’ | 5’-CGGCGCTTCTCGATGATGCC-3’ |
| *Gucy1a3* | NM_021896.5 | 5’-GCCAGCGGAGCAAAGACACC-3’ | 5’-GGTGTTCCTGGAGCTTGGC-3’ |
| *Peg3* | NM_008817.2 | 5’-gcctcagaatccaattcagg-3’ | 5’-tcttcggcaagctggact-3’ |
| *Brd4* | NM_020508.3 | 5’-aaaactccaaccccgatgag-3’ | 5’-gaaccagcaatcacgtcaac-3’ |
| *Zc3hav1* | NM_028421.1 | 5’-tgctctgaccctgttttcttct-3’ | 5’-tcctcatcctggttattgagc-3’ |
| *Siglech* | NM_178706.4 | 5’-ctgcttcttgggatctgctt-3’ | 5’-tgtgtgttgctggtctctcc-3’ |
| *Ligp* | NM_021792.4 | 5’-tgagtgcatccactggaca-3’ | 5’-cagctgacccatgacttcaa-3’ |
| *Irg1* | NM_008392.1 | 5’-gcttttgttaatggtgttgctg-3’ | 5’-ggcttccgatagagctgtga-3’ |
| *Il1b* | NM_008361.3 | 5’-CGAGGCCTAATAGGCTCATCTGG-3’ | 5’-GCCCAGGTCAAAGGTTTGGAAGC-3’ |

**Table S1:** Primer pairs specific for the exclusive detection of *Mus Musculus* genes with accompanying gene symbol and NCBI accession number.
